# Sleep Monitoring of Children With Nocturnal Enuresis: A Narrative Review

**DOI:** 10.3389/fped.2021.701251

**Published:** 2021-09-30

**Authors:** Binbin Zhu, Kun Zou, Jianhua He, Xueqin Huang, Weichao Zhu, Ahmad Khaled Ahmad Harb, Jianhua Wang, Aiping Luo

**Affiliations:** ^1^Department of Anesthesia, The Affiliated Hospital of Medical School of Ningbo University, Ningbo, China; ^2^Department of Electrophysiology, The Affiliated Hospital of Medical School of Ningbo University, Ningbo, China; ^3^Department of Pediatric Surgery, The Affiliated Hospital of Medical School of Ningbo University, Ningbo, China; ^4^Zhejiang Key Laboratory of Pathophysiology, Medical School of Ningbo University, Ningbo, China; ^5^Department of Radiology, The Affiliated Hospital of Medical School of Ningbo University, Ningbo, China

**Keywords:** nocturnal enuresis, arousal disorder, polysomnography, EEG, sleep monitoring

## Abstract

The purpose of this article is to provide a succinct summary of the sleep monitoring efforts that have been used in nocturnal enuresis (NE) and an overview of the knowledge that has accrued. This is not intended to be a comprehensive review, but rather is intended to highlight how polysomnography (PSG), a common sleep detection tool, has contributed to our understanding of NE, as arousal disorder is considered to be one of the important mechanisms. The authors have organized this report by analysis and display of different ingredients of PSG, starting with comparing the electroencephalogram (EEG) of controls and the enuretic children and then moving to evaluation of respiratory patterns of NE and comorbid disease obstructive sleep apnea (OSA). In addition, the authors' goal is to better understand the mechanism of NE by integrating various levels of sleep monitoring; those sleep-related clinical scale scores for NE are presented to date. Finally, we propose further research of NE to explore the microstructure alterations *via* PSG combined with EEG–fMRI or to use novel technology like portable device internet and deep learning strategy.

## Introduction

Nocturnal enuresis (NE), also known as bedwetting, is a common disorder in children and adolescents. It is generally considered that enuresis before 5 years old is a “normal” physiological phenomenon. However, from the age of 6 years, NE is regarded as a disease that needs to be addressed (1, 2), as most children attain nocturnal urinary control by the age of 5 (2). This may affect the growth and development of the child's brain and psychology, which can eventually evolve into a series of psychological problems (3). Domestic research shows that the incidence rate is 11.8% at 5 years old, and the overall incidence rate is 4.07% between 5 and 18 years old (4). However, the incidence of children with enuresis in China is increasing (5), especially primary NE (7.30% in 2017 vs. 4.07% in 2006). NE has obvious genetic susceptibility and genetic tendency (6), and it is speculated that excessive use of baby diapers contributes to the development of NE (7, 8). Albeit 15% of children spontaneously remiss every year, about 2% of the patients have a poor response to treatment, and the course of the disease can be extended to adulthood (9).

Numerous results showed that there were significant differences in the scores of anxiety, depression, emotional disorder, attention deficit hyperactivity, attention problems, disobedience to instructions, aggressive behavior, and post-traumatic stress disorder between NE patients and healthy controls (10, 11). At present, it is believed that NE may be closely related to abnormal bladder function, excessive production of urine during sleep, and arousal disorder after bladder filling (12), but the exact pathophysiology is still unclear. It is worth noting that children with enuresis could not wake up from sleep when their bladder is full, resulting in NE (13). Therefore, arousal disorder may be the key mechanism among the various etiological hypotheses of enuresis.

The aim of this review is to highlight possible mechanisms of NE from studies focused on polysomnography (PSG).

We searched the MEDLINE/PubMed, ClinicalTrial.gov, and Cochrane Library databases for relevant articles published from 1955 to 2021. The reference lists of all retrieved articles were hand-searched. Studies were included if either PSG or other means of sleep monitoring of NE were mentioned; the characteristics of EEG and respiration patterns are discussed to facilitate a better understanding of NE. In the end, alternative sleep monitoring is introduced to inspire future research.

## Research progress of PSG on NE

### Onset Time and Sleep Phase of NE

Half a century ago, Dr. Ditan and Dr. Blinn were the pioneers who used PSG to identify that NE is not a form of epilepsy and is unrelated to epilepsy (14). Numerous studies have evaluated the sleep architecture and characteristics in children with NE after that. At present, there is no consistent study on the onset time of NE. An early study has reported that the first hour after sleep is a period of a high incidence of NE (15), yet some studies argued NE occurred more often in the second and third sleeping cycles (16). Others reported incidence of NE at 2–3 a.m. (17), while some reported it at all times of the night (18). In other words, there used to be no consensus on the time of NE.

In recent years, researchers have suggested that enuresis usually took place in the N2 phase [a phase in non-rapid eye movement (NREM)] and “deep sleep phase” (12, 17, 19). Other studies point out that enuresis may occur during the sleep phase transition period (N3 → N2 or N2 → N1). When the stimulation of urination signal appears, the sleep spindle wave and δ wave in EEG recorded do not decrease, indicating that the child is not awake (20). These studies show that enuresis in children may occur in the non-REM sleep period, especially during the sleep transition. Sleep fragmentation might contribute to the increase of slow-wave activity [indicated by the increase of cyclic alternating pattern (CAP) rate in stage N3 sleep]. Moreover, it explains the higher arousal threshold (indicated by a decrease of phase A2 and A3 indexes) linked to an increased sleep pressure (19).

On the other hand, although enuresis episodes in stage N1 sleep and REM sleep are rare, Dr. Collier and his colleagues found that enuretic children have a larger variation in their REM sleep and sleep efficiency during a wet night when compared with non-bedwetting peers (21), which supported the findings by Ritvo et al. (22) and Bader et al. (23).

Considering the heterogeneity of various studies and significant intraindividual variability, the time spent in different sleep stages differs, and the distribution of enuresis episodes in the different stages can be the result of pure coincidence, so the sleep periods of enuresis may need further clarification.

### EEG Changes During NE

Enuresis occurs during sleep, and an EEG can show different characteristics in different sleep phases and transition between two sleep phases. Sleep–wake transition includes three electroencephalogram (EEG) stages: the first stage is the change of background frequency, with the appearance of a characteristic K-complex wave. In the second stage, there is extensive monotonous θ or δ activity in the anterior part of the head, while the rhythmic activity of the posterior head decreased accompanied by limb activity artifact. In the third stage, there is semi-rhythmic δ activity in the posterior head. Cortical arousal can occur during stages 2 and 3 and may return to REM sleep at any time. In addition to α rhythm or rhythmic θ activity, background activity can also be widely suppressed (24). EEG of enuretic children showed dynamic changes during enuresis. Before enuresis, EEG could show changes similar to sleep–wake transition and last until enuresis, such as rhythmic slow waves lasting for 15–40 s, 6–7 Hz intermittent rhythmic θ in NREM, and 2–3 Hz δ in REM. It is worth noting that the above changes of EEG may not be accompanied by enuresis, or it may be under exogenous or endogenous stimulation or sleep phase transition. During the process of enuresis, the corresponding changes of body signals such as respiration, heart rate, and limb activity may occur along with the dynamic changes of EEG (25). Therefore, it is speculated that for the occurrence of NE to take place, it may require the bladder to be in the process of filling, the abnormal activity of bladder detrusor and urethral sphincter muscles, the disorder of autonomic nerve activity regulation, abnormal EEG arousal activities, and other related factors. Table 1 compares the EEG changes in NE and normal controls of school-age children.

**Table 1:** The comparison of EEG changes in NE and normal controls of school-age children during the night.

	**Patients with nocturnal enuresis**	**Normal controls**
Total sleep time	Decreased (26)	No difference (19)
REM sleep	Higher variability (21)	Not increased
Depth of sleep	Light sleep but difficult to wake up during enuresis (12). Deeper depth in children with NMNE (27)	Normal sleep circle
Wake %	Frequent in PNE	Rarely
Sleep efficiency	Lower sleep efficiency especially with comorbidity (21)	No difference between primary monosymptomatic NE with normal controls (19)
Abnormal EEG detected	Show changes similar to sleep–wake transition before enuresis; increased delta power	Not detected
Cortical arousal index (CAI)	A decreased CAI in NREM (19) An increased CAI in refractory patients (28)	Fewer sleep fragmentation
Cyclic alternating pattern (CAP)	Increase in CAP rate during N3 (slow wave activity)	No significant disruption

### Evaluation of Respiration Patterns at Night

NE is often associated with obstructive sleep apnea (OSA), especially in obese children, and some OSA patients have nocturnal symptoms including enuresis. However, the presence of habitual snoring is insufficient for the diagnosis of OSA because many children who snore do not have OSA. Thus, PSG and a sleep expert are needed for accurate diagnosis (29).

Children with obstructive apnea/hypopnea index (AHI) ≥ 2 episodes/h were considered as OSA. In the study by Shafiek et al., 68.5% of school-age children who presented with NE had OSA, with a median obstructive AHI of 6.1 (3.7–13.2) episodes/h. On the one hand, the result shows the necessity of screening OSA for NE sufferers; on the other hand, it calls for further analysis since OSA and NE share a complex interaction. Table 2 lists some features and compares OSA and NE.

**Table 2:** The comparison of NE and OSA *via* sleep monitoring.

	**NE**	**OSA**	**NE+OSA**
EEG evaluation	REM time is longer; sleep fragmentation increases in children with refractory enuresis (29)	Diminished amounts of slow waves and REM sleep; sleep fragmentation (30)CAP increases (31)	Increase in CAP rate in stage N3 sleep (19)
Apnea–Hypopnea Index (AHI)	Higher AHI and greater variance in both oxygen and carbon dioxide measures compared with control (32)	Higher AHI or respiratory disturbance index (RDI) than non-OSA	Higher AHI
Hemodynamic data	Higher MAP levels during sleep compared to controls (33)	Increased BP and BP variability (34)	High nocturnal blood pressure levels (35)
CPAP effectiveness	NO STUDY	More stable EEG pattern after CPAP	Alleviate OSA and enuresis in adults with both conditions (36)
Tonsillectomy and adenoidectomy/ adenotonsillectomy effectiveness	Improvement of PNE in more than 60% of patients, with complete resolution rates in excess of 50% (37)	Part of PSG parameters improved for mild OSA but no improvements in objective attention (38); prominent improvement in AHI and other polysomnography data especially in non-obese children (39)Small difference in AHI changes compared with wait group (40)	No effect on resolution of PNE (41)
Desmopressin	6.1–22.4% response rate in PNE (42)	NO STUDY	Relapses on discontinuation of desmopressin (43)

### Observation of Limb Movement

Periodic limb movement disorder (PLMD) is characterized by periodic episodes of repetitive and highly stereotypical limb movements during sleep, as documented on PSG, and associated with sleep disturbance or daytime dysfunction. The movements usually involve an extension of the great toe and partial flexion of the ankle, knee, and sometimes hip. When these movements are associated with repetitive partial arousals or awakenings, sleep is fragmented. In addition to the movements, affected children have daytime problems, often including diminished attention.

Studies have found that periodic leg movement increases in children with refractory enuresis (29) and patients with monosymptomatic NE and polyuria (28). Periodic leg movements are associated with enuresis (20). Compared with children who do not suffer from NE, enuretic episodes in children with NE have a longer duration and more times of restless legs (21).

## Sleep Evaluation Batteries and Questionnaires for NE

The Pittsburgh Sleep Quality Index (PSQI), which is a self-report questionnaire composed of 19 items assessing sleep quality and disturbances over a 1-month interval, is used to identify people with poor sleep quality. The PSQI includes subscales on sleep duration and daytime dysfunction that can be useful to detect and follow insufficient sleep. Table 3 lists the scales commonly used in children's sleep assessment.

**Table 3:** Comparison of widely used batteries for sleep assessment of NE patients.

**Name of battery for NE**	**Advantage**	**Disadvantage**
PSQI	PNE was significantly correlated with the PSQI total score (sleep quality) (5)	Subjective and not likely to be useful as screening measures for PSG (44)
Epworth Sleepiness Scale	Initial assessment of sleep	Not objective
Sleep clinical record score	Multi-dimensional and validated (45)	Complicated to calculate
The sleep timing questionnaire	Reasonable test–retest reliability	Patient adherence with sleep diaries is a concern

## Alternative Sleep Monitoring in NE

### The Application of a Portable Sleep Monitor to Diagnose NE

PSG in the laboratory is the gold standard to diagnose OSA and NE traditionally. Now, there are other alternative methods, as the PSG device has multiple channels and is not so user-friendly. The rise of wearable devices like Fitbit Charge 2, a wrist-worn sleep tracking device, could detect REM sleep (21), which is a useful supplement to the home sleep apnea testing (HSAT) device. Actigraphy has been validated against PSG and shown to provide a reasonable estimate for patterns of sleep vs. wakefulness in children and adults. The advantage of actigraphy over PSG is that it captures multiple days of data from the home environment. Actigraphy is typically used by sleep medicine specialists as part of a comprehensive analysis of sleep–wake patterns, as well as to monitor response to interventions. However, since HSAT devices have fewer channels, some technical errors such as motion artifacts may be encountered, and according to the categorization rule of SCOPER (46), different physiological parameters need to be integrated to draw a whole picture for NE.

### Application of Internet and Deep Learning

The Internet of Things (IOT) architecture enables real-time monitoring of human physiological parameters, combined with diagnostic algorithms to provide early warnings of abnormal data (47). Studies led by Ma et al. suggested that deep learning methods achieve robust sleep staging results of both portable and in-hospital EEG recordings. In addition, it may allow for more widespread use of ambulatory sleep assessment tests across a variety of clinical conditions, including neurodegenerative disorders (48). The diagnostic accuracy of a novel algorithm for the estimation of sleep stages and disease severity in patients with breathing-disordered sleep is based on actigraphy and respiratory inductance plethysmography (49).

### The Combination of EEG–fMRI on the Mechanisms of NE

The hypothalamus is an important region in the brain that is responsible for promoting arousal. Its dysfunction could result in the loss of bladder control during sleep (12, 50). Zhang et al. carried a resting-state fMRI study and reported the presence of four brain regions found with a reduced connection efficiency to the thalamus: the posterior lobe of the cerebellum, the frontal lobe, the parietal lobe, and the precentral gyrus. It can be concluded that these relevant regions might induce an arousal disorder and therefore lead to PNE (51).

Children with PNE had a higher percentage of total sleep time in light sleep and a higher arousal index compared with controls. Abnormal thalamocortical functional connections were detected in the lateral prefrontal cortex, medial prefrontal cortex, and inferior parietal lobule during light NREM sleep. Abnormal intra-thalamic FCs were also detected during light NREM sleep among the motor, occipital, prefrontal, and temporal subdivisions of the thalamus (52). Although such kind of nice work is difficult to carry out, as it is difficult to perform a randomized multi-center large sample study, it is of great significance to elaborate the mechanism, and future work is needed to better connect the parallel changes in PSG and EEG–fMRI.

## Discussion

NE exerts a great impact on children's psychology, family, and social interactions. At present, patients with suspected enuresis are encouraged to undergo a thorough evaluation to comprehensively evaluate urinary signs and symptoms and eliminate the root cause of diurnal incontinence (53). Desmopressin as first-line therapy for NE could reduce the enuresis frequency (54) and is reported to be more effective for the kids suffering from primary monosymptomatic nocturnal enuresis (PMNE) (55). However, there are difficult moments in the clinical setting when monotherapy, or even combined with behavioral therapy, is not effective. While some studies suggest that anticholinergic drugs, energetic drugs, imipramine, or sertraline may be useful for some drug-resistant patients (56, 57), the challenges to identify the causes of NE patients remain.

To cope with the challenges, systemic evaluation of NE patients more than 6 years old is recommended, including the detailed comprehensive medical history collection (establishment of enuresis diary), physical examination, and auxiliary examination (urinary ultrasound and urodynamics), as well as the detection of plasma AVP and/or plasma copeptin levels (1, 58). On the other hand, in cases of negative findings from laboratory and other exams, PSG may be helpful in further investigation.

Based on our existing literature review and daily practice, PSG is suitable for the following application scenarios: First, PSG could facilitate pediatric urologists to identify children with NE combined with OSA and formulate an effective diagnosis and personalized treatment plan, for example, surgical treatment of airway obstruction may not be encouraged for patients with OSA and PNE, as it had no effect on resolution of PNE (41). Secondly, PSG offers the pediatric physician an opportunity to observe the sleep microstructure and use the EEG CAP to interpret the stability of sleep, especially for those kids with refractory enuresis; a recent CAP-related study showed that the increase of A1 ratio in N3 indicates NREM sleep instability, and the decrease of A2 and A3 percentages in N2 and A3 index in N3 may be related to the high arousal threshold (59). Thirdly, PSG data could be bundled with other parameters, such as bladder urinary volume, heart rate, sleep stage, and periodic limb movement in sleep (PLMS) (20), to estimate when the enuretic incident occurs and thus lay a solid foundation for the design of pre-void wearable alarm in the future.

In addition, as portable or wearable PSG home testing devices become more and more available, pediatric urologists can extract the characteristics of sleep data from the accumulating and expanding data through artificial intelligence analysis, to evaluate the effect of a certain drug and/or behavioral therapy more accurately.

## Conclusion

Distinguishing subtle changes of PSG is crucial in differentiating NE from its comorbidities. Although not the first-line examination for NE patients now, PSG holds promise to precision medicine; pediatric urologists can speculate the exact time of enuresis, predict and classify NE in advance, and offer individualized treatment for NE; and PSG may be the direction of research in the treatment of enuresis.

## Author Contributions

BZ, KZ, and AA equal contribution for the literature search, writing, and correcting of this review article. JH conceived the writing of this article. XH, JW, and WZ offered important advice on the revision of the essay. AL searched the literature according to the review, took minutes of the meeting, and participated in the revision of the article. All authors contributed to the article and approved the submitted version.

## Funding

This work was supported by the Joint Fund of Hospital and Medical School of Ningbo University (No. 201804), the Zhejiang Provincial Natural Science Foundation (No. LY18H280004), Ningbo Science and Technology Bureau (No. 2019A610272, No.2020F027), Medical Health Science and Technology Project of Zhejiang Provincial Health Commission (NO. 2021KY318), and the Major Special Project of Science and Technology Innovation 2025 of Ningbo Science and Technology Bureau (No. 2019B10035).

## Conflict of Interest

The authors declare that the research was conducted in the absence of any commercial or financial relationships that could be construed as a potential conflict of interest.

## Publisher's Note

All claims expressed in this article are solely those of the authors and do not necessarily represent those of their affiliated organizations, or those of the publisher, the editors and the reviewers. Any product that may be evaluated in this article, or claim that may be made by its manufacturer, is not guaranteed or endorsed by the publisher.

## Abbreviations

PSG: polysomnography
MAP: mean arterial pressure
CAP: cyclic alternating patterns
PNE: primary nocturnal enuresis
NMNE: non-monosymptomatic enuresis
AHI: apnea-hypopnea index
NREM: non rapid eye movement
HRV: heart rate variation
EEG: electroencephalogram.
